# Weather impact on ambient air pollution and its association with land use types/activities over 5,572 municipalities in Brazil

**DOI:** 10.1016/j.heliyon.2024.e31857

**Published:** 2024-05-24

**Authors:** Francisco Jablinski Castelhano, Weeberb J. Réquia

**Affiliations:** a Geography Department, Federal University of Rio Grande do Norte Natal, Av. Sen. Salgado Filho, S/n - Lagoa Nova, Natal, Rio Grande do Norte, Brazil; b School of Public Policy and Government, Fundação Getúlio Vargas, Brasília, Distrito Federal, Brazil

**Keywords:** Air pollution, Weather penalties, Land use and cover

## Abstract

Quantify the impact of meteorological changes on air pollution levels is the aim of numerous recent studies. However, there is still a lack of investigations assessing the influence of land use/activities on the relationship between climate and air quality. In this study, we used a two-stage design to estimate the influence of land use types and activities on the association between weather changes and air pollution (PM_2.5_, NO_2_, SO_2_, O_3_) over 5572 municipalities in Brazil. To calculate the influence of recent weather change on air pollution concentration for each municipality, we used the “weather penalty” concept. This approach considers differences in linear trend coefficients between two generalized additive models. Then, using quantile regression, we estimated the effect of land use types and activities (8 variables related to transportation, energy generation, and land use) on weather-related increases in ambient air pollution. We found that an increase in PM_2.5_ was associated to recent weather changes in most municipalities (average increase of 0.07μg/m^3^per year) and a decrease in NO_2_ in most municipalities (average decrease of 0.0003 ppb per year). O_3_ and SO_2_ had more intense increases associated with weather changes in the North region. Our findings suggest the most robust positive associations between weather penalties on PM_2.5_ and areas with non-clean energy and oil refineries (average increase of 0.006μg/m^3^per year and 0.04μg/m^3^per year, respectively). We also found positive associations between Pasture areas, urban areas, and transportation and the weather penalties of this pollutant. In contrast, forest areas were negatively associated with PM_2.5_ penalties. We also found that oil refineries, urban areas, and transportation significantly positively influenced weather penalties for SO_2_ and O_3_. Overall, the study highlights the importance of considering the influence of land use types and activities on weather-related changes in ambient air pollution.

## 1 INTRODUCTION

Weather conditions are associated with significant impacts on air quality [[1], [2], [3], [4]]. Several investigations have suggested that the influence of weather over air pollution concentrations have a significantly time and spatial variability due to the different types of ambient air pollutants and the complex process related to their formation and removal. For example, tropospheric ozone (O_3_) primarily results from photochemical reactions between nitrogen oxides (NOx) and organic compounds hydrocarbons in the presence of sunlight [5]. Besides the influence of these photochemical reactions, O_3_ formation is favored by high temperatures, low humidity, and wind speed [6]. High temperatures can also increase oxidation and production of sulfate particles in the atmosphere [7,8]. In addition, other studies have shown that fine particulate matter (PM_2.5_) is correlated with wind speed (advection and turbulence favor PM_2.5_ dispersion) and precipitation (wet deposition and removal favor PM_2.5_ decrease) [9,10].

Recent studies have defined a Weather penalty as the influence of long-therm weather changes on ambient air pollution [11]. This concept can be seen in a study conducted in the U.S, that revealed that long-term weather changes for 30 years (1988 and 2018) were associated with an increase in nitrogen dioxide (NO_2_), which classifies as a positive weather penalty, mainly in cities with substantial traffic emissions, including Detroit, New York, Houston, and Philadelphia [12]. Other investigations have found high concentrations of PM_2.5_ in both urban and rural areas [13,14]. Their finds suggests that, besides the influence of the spatiotemporal conditions on the association between weather changes and air pollution, information on land use types and activities also plays an essential role. Spatial factors such as volume of traffic, distance to highways [15,16], to green areas/and water bodies [[17], [18], [19]], presence and type of industries and power plants, and agricultural activities [[20], [21], [22]] are considered critical to understand the complex spatiotemporal variability of air pollutants concentrations. Similar results were found in a study conducted in Europe, that reported a positive association between PM_2.5_ concentrations and the density of urban constructions, while water bodies and green areas presented a negative correlation [14].

In our recent study in Brazil, we estimated the weather penalties of five air pollutants (NO_2_, SO_2_, CO, O_3_, and PM_2.5_) in the Brazilian regions [23]. PM_2.5_ was the most impacted by the weather changes, revealing positive weather penalties in all Brazilian regions while SO_2_ and CO had different penalties (direction of the association) depending on the region. However, the scope of this study was limited to the regional level, as we estimated the impact of weather changes on air pollutants levels by using a meta-analysis in a total of 5 regions in Brazil. This coarse-scale limits the interpretation of the results in the context of the influence of land use/activities on the relationship between climate and air quality. Also, this regional analysis may underestimate or overestimate the regional trends given that the spatial distribution of the air pollution concentrations, and climate conditions are not homogeneous. This present contribution addresses these limitations by expanding our previous analysis, quantifying the past weather-related changes (2003–2018) in ambient air pollution concentrations over 5572 municipalities in Brazil.

Then, we assessed the influences of land use types and activities on the association between weather changes and air pollution.

## 2 Materials and methods

### 2.1 Data

#### 2.1.1 Air pollution

We estimated the impacts of weather changes on four ambient air pollutants, including PM_2.5_ (μg/m^3^), NO_2_ (ppb), O_3_ (ppb), and SO_2_ (μg/m^3^). These are the most relevant pollutants concerning health effects and are suggested to be impacted by recent climate changes [12,23,24].

We used daily average concentrations of these pollutants for the period 2003–2018 from the Copernicus Atmosphere Monitoring Service (CAMS)-Reanalysis (from the European Centre for Medium-Range Weather Forecasts – ECMWF). The data was retrieved at a spatial resolution of 0.125° (approximately 12.5 km), covering all Brazilian territory, and was aggregated at the municipality level, considering only the mean concentration for the headquarters of each municipality. This aggregation was important for the study, since the municipalities in Brazil have a certain level of autonomy over their territories to develop specific policies concerning their urban mobility, land use restrictions, environmental issues, economic factors, and other parameters that can rely on air pollution policies. The municipalities represent the smallest territory in the Brazilian political system, with a total of 5572 municipalities. In the supplementary materials, we show the spatial distribution of the Brazilian municipalities.

The validation of the CAMS global model for O_3_ and NO_2_ was made through a comparison with surface data from the World Meteorological Organization (WMO) Global Atmosphere Watch (GAW) program averaged globally. This comparison supports that the data from CAMS is highly correlated with monthly mean values and the seasonal variability of the pollutants. Concerning O_3_, means CAMS reanalysis data agrees with the surface data to within 10 % for most years with a smaller bias after 2003 [25].

The validation of PM_2.5_ data from CAMS was based on a comparison with ground observations of the Aerosol Robotic Network (AERONET) spread worldwide, including 27 stations located in every region of Brazil, measuring spectral Aerosol Optical Depth (AOD) with ground-based sun photometers. The comparison between CAMS data and the AERONET in South America revealed a small bias of approximate −0.006 ± 0.128, validating their use [25].

#### 2.1.2 Weather

We used meteorological data from the ERA-Interim model, consisting of a global atmospheric reanalysis performed by the ECMWF. For this study, we gathered a meteorological dataset consisting of four variables: surface temperature (°C), precipitation (mm), relative humidity (%), and wind speed (m/s). Weather data was retrieved at a temporal resolution of 6 Hours and a spatial resolution of 12.5 km and was aggregated into a daily scale by the municipality.

#### 2.1.3 Land use

We used land use and cover data from the Brazilian Annual Land Use and Land Cover Mapping Project – MapBiomas (https://mapbiomas.org/). This data was produced from the pixel-by-pixel classification of Landsat satellite images, with a resolution of 30 m for the year 2018. Among the several land use/cover classes considered in the MapBiomas, we selected those that are related to air pollution, such as i) Forest areas, which includes forest and savanna formation, mangrove areas, and forest plantation - studies have suggested that green spaces are negatively correlated with air pollution [13,14]; ii) Pasture areas, representing original vegetation transformed into pasture areas for cattle feed - a positive correlation between pasture areas and air pollutant concentrations, specifically O_3_, NO_2_ and PM_2.5_ is suggested by the literature [13,26,27]; iii) Agriculture areas, representing, temporary crop, soybean, sugar cane, other temporary crops, perennial crop, and mosaic of agriculture - mostly due to fires and the use of pesticides, agriculture areas are also positive correlated to air pollution concentrations [13,28]; and iv) Urban areas, representing all urban infrastructure - due to the concentration of emission sources such as industries and vehicle traffic, high density of buildings and lack of green areas, these areas are usually positively correlated to air pollution concentrations [29]. Each one of these land use categories was measured in hectares and aggregated by municipality.

#### 2.1.4 Energy sources

We obtained data on energy sources from the MapBiomas project, concerning the information for the year 2018. This data contains information on i) Clean energy, which represents the sum of wind, hydroelectric, solar, and nuclear power plants; and ii) Non-Renewable Energy, representing the sum of fossil fuel and biogas power plants; iii) Oil refineries, which we considered the summary of oil refineries in each municipality.

#### 2.1.5 Transportation data

We accessed transportation data from the National Traffic Department of Brazil (DENATRAN). We accounted for the mean vehicle fleet for each municipality for the period 2003–2018.

### 2.2 Analyses

We used a two-stage design in this study. First, we calculated the influence of recent weather change on each air pollutant concentration by using the concept of “weather penalty”. Then, in the second stage, we estimated the effect of land use types and activities on weather-related increases in ambient air pollution. We accounted for eight factors divided into three groups: land use (agriculture area, forest area, urban area and pasture area), energy sources (clean energy sources, non-clean energy sources and number of oil refineries), and transport (total vehicular fleet). Details of each stage are described in the following sections.

#### 2.2.1 LONG-TERM impact of weather change on air pollution

In the first stage of this study, we used the “weather penalty” technique [11] to calculate the influence of long-term weather changes on air pollution. We perform this approach by measuring the differences between the linear trend coefficient (β values) between two Generalized Additive Models (GAM) controlled for temporal terms, including yearly, monthly, daily and weekday variation. The first model, however, was adjusted by the weather variables (Wind speed, humidity, temperature, and precipitation), while the second model was unadjusted, which means that the adjusted model is controlled for the weather parameters, while in the unadjusted model, the weather variables are excluded.

Several recent studies used this approach to estimate the impact of weather over air pollution in different regions such as USA [11,12], Spain [24] and Brazil [23,30,31].

Equations (1), (2)), reveals the unadjusted and adjusted models respectively.

(1) Yi,j,p=βo+βunadjustedYeari,j,p+γmonthi,j,p+δweekdayi,j,p+εdayi,j,p

(2) Yi,j,p=βo+βadjustedYeari,j,p+γmonthij+δweekdayi,j,p+εdayi,j,p+s1(temp)ij+s2(prec)ij+s3(ws)ij+s4(rh)ij

where *Y* represents the daily average concentration of the ambient air pollutant *p* (PM_2.5_, NO_2_, O_3_, and SO_2_) in the municipality *i* on date *j*; *β*_*0*_ is the intercept of the GAM model; *β*_*unadjusted*_ and *β*_*adjusted*_ represent the linear weather-unadjusted and adjusted pollutant trends, respectively, expressed in μg/m^3^ per year for SO_2_ and PM_2.5_ and in ppb per year for O_3_ and NO_2_; *δ* and *Ɛ* are the vectors of coefficients that explain monthly, and weekday variability within the time series, respectively; and *s1, s2, s3*, and *s4* are the default smoothing spline functions from the mgcv R package [32], which was used for considering the nonlinear relationships between the daily concentration of pollutant *p* and weather variables, including temperature (temp), precipitation (prec) wind speed (ws), and relative humidity (rh), respectively in the weather-adjusted model (Equation (2)).

Note that while the *β*_*unadjusted*_ (Equation (1)) incorporates the weather impacts over the pollutant concentrations, the β_adjusted_ (Equation (2)) removes the influence of inter-annual temperature, precipitation, wind speed and relativy humidty variation over air pollution trends. Therefore, the influence of weather changes during the air pollution time series, or the weather penaltis, for each municipality, are derived through the differences between β_unadjusted_ and β_adjusted_ (β_unadjusted_ - β_adjusted_). Thus, we assume that the recent weather changes are associated with an increase in a specific pollutant level If the penalty calculated is a positive value (*β*_*unadjusted*_ *> β*_*adjusted*_).

Finally, to estimate the confidence intervals of the coefficients previously calculated we applied a bootstrap analysis based on 1000 randomized subsets (pseudo-datasets) of the original dataset for each municipality where the same models described above (equations (1), (2))) were also applied.

After that, we estimated the standard error by obtaining standard deviation from the 1000 estimates in the bootstrap analysis. We used the GAM function from the mgcv package [32] at RStudio to perform the equations mentioned.

#### 2.2.2 Effect of land use types and activities on weather-related increases inambient air pollution

Due to the large heterogeneity of landscapes and social inequality across Brazilian municipalities, the relationships between multiple land use variables and air pollutant concentrations are likely to vary through the distribution of the air pollutants (quantiles of the air pollution concentration) [22]. Therefore, in the second stage of our study, to estimate the relationship between the weather penalties and the land use variables, we used a quantile regression model, which can obtain a more comprehensive picture of the different effects of the independent variables on dependent variables [10,22,33]. While the simple linear model estimates the association between the response variable, in our case, the weather penalties for each municipality, and the predictor variables (for this study each land use, transportation and energy metrics for each municipality) based on the mean of the response variable, a quantile regression estimates the conditional quantiles (or other positions of the distribution) of the response variable. This method makes no assumptions about the distribution of residuals, and it has a distinct advantage for detecting variation effects [34]. The Quantile Regression model was performed in R, with the use of the quantreg package [35]. The equations used for the quantile regression are as follows:

(3) WPi=Xiβθ+uθi,0<θ<1

(4) Quantθ(WPi∨.i)=xiβθ

where WP denotes the dependent variable, in our case, the weather penalty for each pollutant; x is a vector of the independent variables (each land use, transport and energy variable were included at the time in the model); *i* indicates the quantile; and u indicates a random error term, which conditional quantile distribution is equal to zero. *Quant****θ***(*yi* ∨.*i*) represents the θ^th^ percentile of the dependent variable WP, in which for this study we used the 5,10,25,75,90, and 95th percentiles.

The scripts used in the analysis are available at the repository https://github.com/chicozo1989/QuantileRegression.

## 3 Results and discussion

### 3.1 Weather penalties in the Brazilian municipalities

**Fig. 1:**
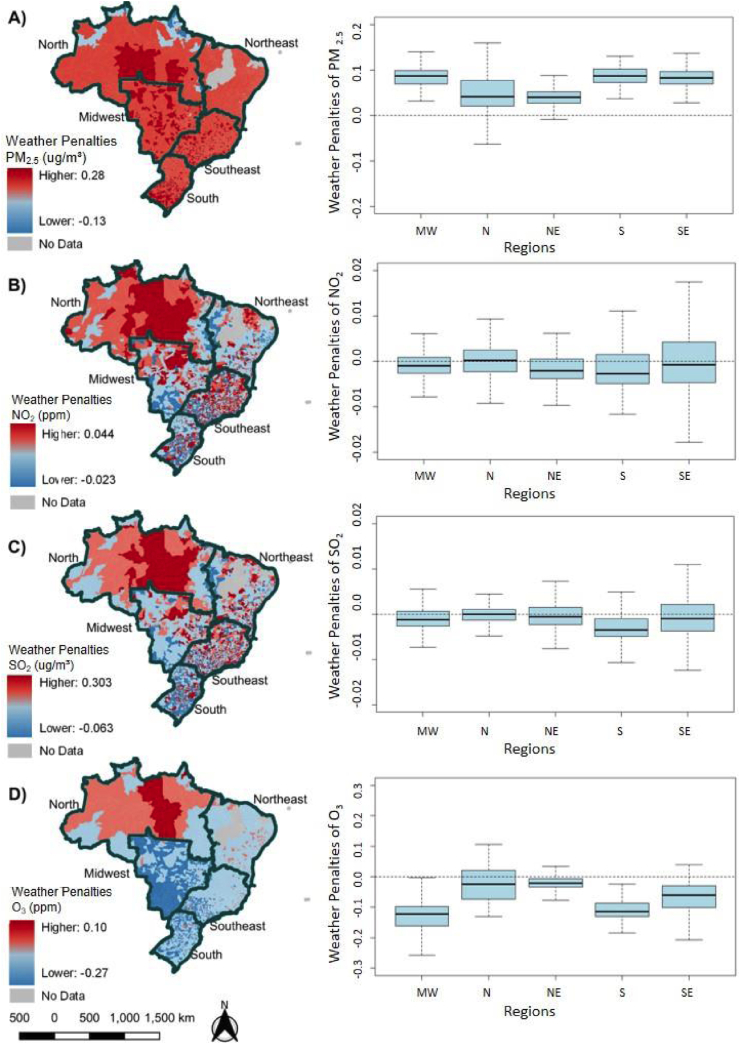
Weather penalties for PM_2.5_ (A), NO_2_ (B), SO_2_ (C) and O_3_ (D) for the period 2003–2018. Note: On the left side, we show the maps with the weather penalties across the Brazilian municipalities. On the right side, we show the boxplots of the weather penalties stratified by region. MW is for Midwest region; N is for the North region; NE is for Northeast region; S is for South region and SE is for southeast region.

Fig. 1 shows the weather penalties for each pollutant nationwide stratified by region and municipality. Our results indicate a substantial variation in the weather penalties depending on the region and the air pollutant.

For PM_2.5_ (Fig. 1, map A), we estimated positive weather penalties in most of the Brazilian municipalities (a total of 5086). This indicates that the long-term weather changes during the study period were associated with an increase in PM_2.5_ in most of the municipalities in Brazil. Although recent studies have reported a decrease in PM_2.5_ during the last two decades, as a direct benefit of public policies to control traffic emissions [36,37], our results suggest that meteorological factors played an important role. If the weather parameters had remained constant between 2003 and 2018, on average, the concentration of PM_2.5_ in Brazil would have decreased even more. The highest weather penalty on PM_2.5_ occurred in a municipality in São Paulo State, located in the Southeast region, with an estimated annual penalty of 0.28 μg/m^3^. The fifty highest weather penalties were all found in municipalities in the state of São Paulo, the most urban and industrial state of Brazil.

For NO_2_ (Fig. 1, map B), different from PM_2.5_, most of the municipalities had a negative weather penalty (3267 municipalities). Previous studies have shown that in the last years, weather conditions (increases in wind speed and precipitation) in Brazil are associated with decreases in NO_2_ [23]. As seen in PM_2.5_, the southeast region had the highest positive weather influence on NO_2_, with an estimated 0.044 ppb/year.

Weather penalties on SO_2_ (Fig. 1, map C) had a similar spatial pattern compared to NO_2_. We found a total of 3270 municipalities (mostly in the Northeast region) with negative penalties on SO_2_. Positive weather penalties were found in 1880 municipalities, mostly in the Southeast and North regions. Our findings agree with recent literature that highlighted decreases in pollutant levels concentrations in South and Southeast Brazil [38,39]. SO_2_ levels concentrations in Brazil are mostly related to fossil fuel burn, which includes thermoelectrical power plants that are an alternative to the hydro-electrical power plants during the dry season, when the first ones are usually turned on [40].

Finally, a municipality in the North region had the highest weather penalty on O_3_ (Fig. 1, map D), with an estimated 0.28 ppb per year. Our results indicate that the top 40 positive penalties were all found in the North region, however this pollutant revealed the least number of positive weather penalties with a total of 442 municipalities, of which 193 of them are in the North region. In contrast, we estimated negative penalties on O_3_ in 4735 municipalities, mostly in the Midwest and Northeast regions. These results found for O_3_ are in agreement with previous studies that also revealed negative impact of weather changes over this pollutant across the country [23]^.^

### 3.2 Energy, transportation, and land use variables in the Brazilian municipalities

**Fig. 2:**
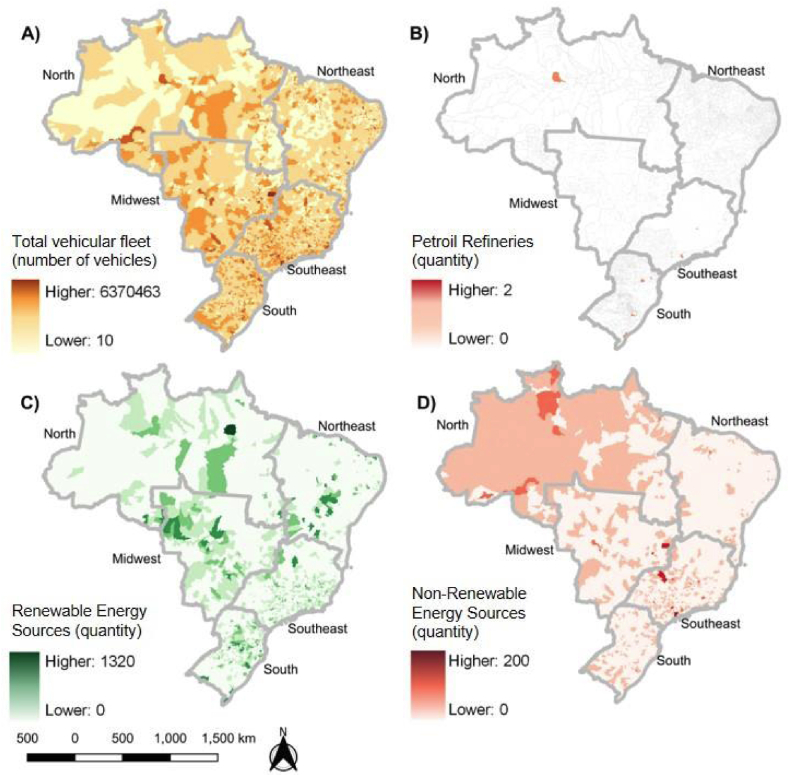
Vehicular Fleet (A), oil refineries (B), clean energy power plants (C), and non-clean energy power plants (D) for the period 2003–2018 per municipality.

Fig. 2 shows the spatial distribution of the energy (clean energy sources, non-clean energy sources, and oil refineries) and transportation (Vehicular Fleet) variables considered in our analyses. The total vehicular fleet (Fig. 2, map A) included more than 67 million vehicles, of which 49.5 % are in the Southeast region (33.6 million), 20.2 % (13.7 million) in the South region, 17.4 % (11.8 million) in the Northeast region. Midwest and North have less than 10 million vehicles, accounting for 5.8 million and 2.8 million, respectively.

The highest concentration of oil refineries (Fig. 2, map B) is in the Southeast region (9 municipalities with oil refineries), followed by the Northeast and South (both with 5 municipalities). The Midwest region does not have any municipality with an oil refinery.

Clean energy sources (sum of wind, hydroelectric, solar, and nuclear power plants) are concentrated over the North region (1463 clean power plants) and Southeast (1088 clean power plants), followed by the Northeast (836 clean power plants), South (831 clean power plants), and Midwest with only 470 clean power plants (Fig. 2, map C). In contrast, there are fewer non-clean energy sources (fossil fuel and biogas power plants) in Brazil (Fig. 2, map D). The Southeast and North are the regions with the most non-clean energy sources, with 1205 and 462 non-clean power plants, respectively.

**Fig. 3:**
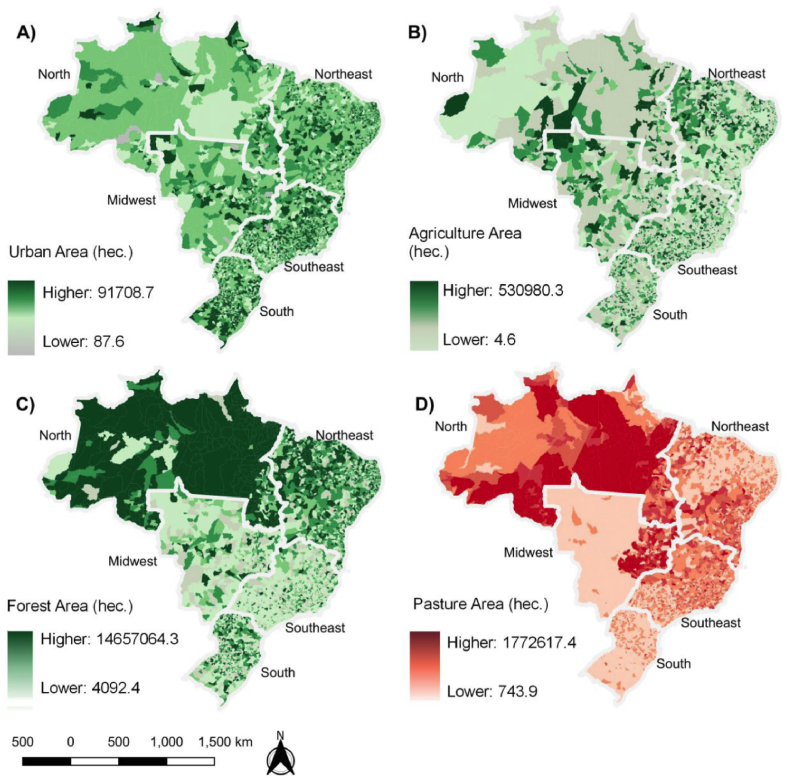
Spatial distribution of land use variables in Brazil in 2018. Note: Urban area (A), Agriculture area (B), Forest area (C), and Pasture area (D).

Finally, Fig. 3 shows the spatial distribution of the land use variables. Southeast is the region with the highest amount of urban areas, reaching up to 1,61 million hectares. North, where the Amazon is located, is the least urbanized region in Brazil, with approximately 0.34 million hectares of urban areas (Fig. 3, map A).

Agricultural areas (Fig. 3, map B) had the highest concentration in the Southeast (8.68 million hectares) and Northeast (8.28 million hectares) regions, followed by the South (4.12 million hectares) and Midwest (2.28 million hectares) regions. The North region had the lowest concentration of agricultural areas, with 2.28 million hectares.

Forest areas (Fig. 3, map C) are highly concentrated over the North region (475.3 million hectares), where most of the Amazon Forest is preserved. The Northeast region, specifically the West part, has 236.4 million hectares of forest areas, followed by the South region (61.9 million hectares) and the Southeast region (28.6 million hectares). The Midwest region has the smallest forest areas, with 10.4 million hectares.

For the Pasture area (Fig. 3, map D), the North and Midwest regions have the highest concentration, with 42.3 and 32.4 million hectares, respectively. Southeast and Northeast regions have similar patterns, with 29.6 and 29.9 million hectares of pasture area, respectively.

### 3.3 Influences of the land use types/activities on the weather penalties

#### 3.3.1 p.m.2.5

**Fig. 4:**
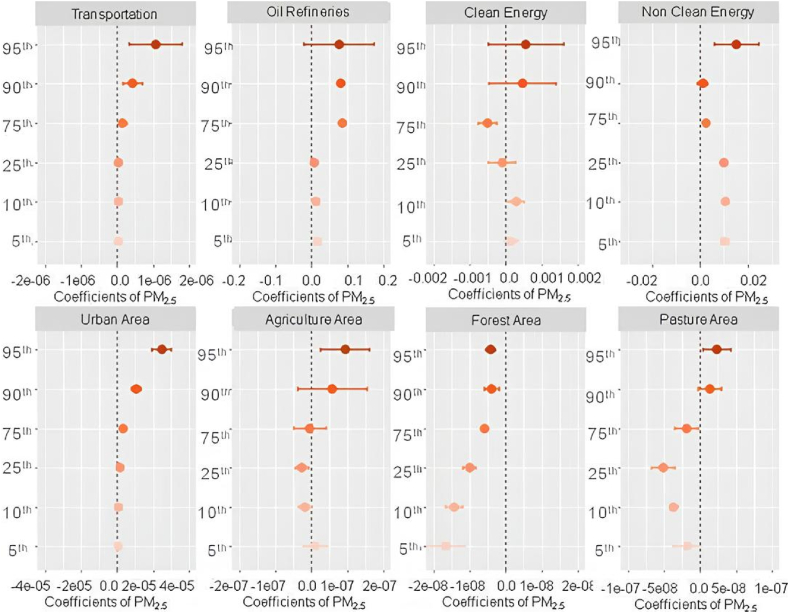
Quantile regression coefficients for weather penalties and land use types/activities factors over PM2.5 for the period 2003–2018.

Thus, robust positive associations with weather variation on PM_2.5_ were observed in locations with agricultural activities, non-clean energy, oil refineries, pasture, urban areas, and high vehicular fleet (Fig. 4). Overall, these associations were higher at the highest percentile (95th) of the weather penalty on PM_2.5_. In contrast, negative associations were observed for forest areas (in all percentiles), clean energy (quantile 75th), and pasture areas (< quantile 75th).

The influence of oil refineries, traffic, and non-renewable energy sources over the weather penalties on PM_2.5_ is supported by the literature showing that fossil fuel burning, which are related to all those variables, are the main source of particulate matter emissions [29,[41], [42], [43]]. Same factors can explain the positive coefficients found for urban areas, which are commonly the places with higher vehicles concentration and also, concentrates other small and domestic sources of biomass burning such as wood stoves that can impact the PM concentrations as seen in other studies from India, and Brazil [[44], [45], [46], [47]]. The negative associations observed in forest areas and pasture areas can be attributed to the deposition process of particulate matter, and less soil suspension due to the preservation of natural land covers, which leads to a reduction in PM_2.5_ concentrations. Studies developed in both natural and urban areas have revealed the role of the stomata of the leaves in the particulate matter absorption, agreeing with our findings [44,[48], [49], [50], [51]].

#### 3.3.2 NO_2_

Nitrogen Dioxide is a gas resulting from fossil fuel burning. Light and radiation, however, can promote the photolysis process on this pollutant, resulting in the dissociation of the NO_2_ molecule into NO and atomic oxygen. The atomic oxygen can react with the O_2_ from the atmosphere and with volatile organic compounds (VOC), resulting in the formation of O_3_ [52,53].

**Fig. 5:**
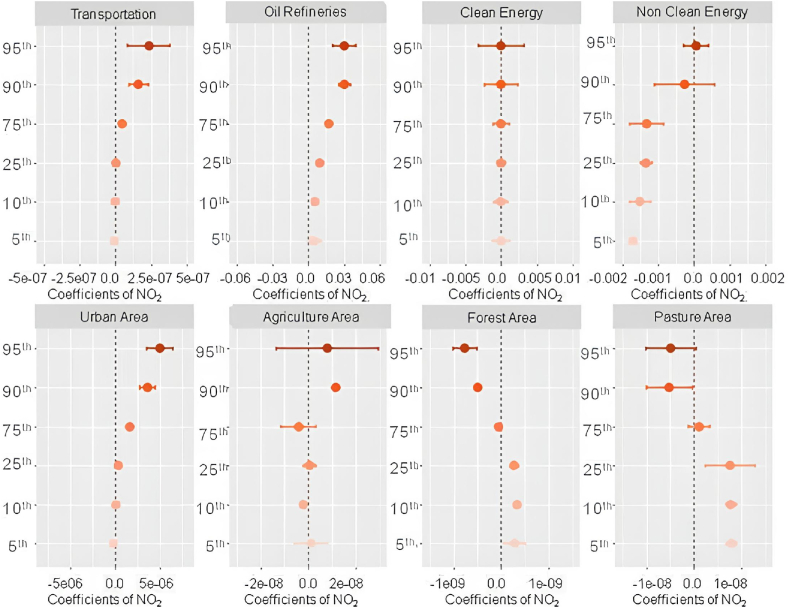
Quantile regression coefficients for weather penalties and land use types/activities factors over NO2 for the period 2003–2018.

Our findings suggest that transportation, oil refineries, and urban areas are positively associated with penalties on NO_2_, and this association becomes stronger with increasing levels of the weather penalty on NO_2_ (Fig. 5). These results are consistent with previous studies that have identified that local sources, such as transportation as a major source of NO_2_ emissions in urban areas, followed by industrial sources and domestic sources from heating or cooking systems [[44], [45], [46],^54^,^55^].

Additionally, our findings suggest that agricultural areas may also contribute to weather penalties on NO_2_, particularly at higher levels of the weather penalty. This may be due to the use of fertilizers and other agricultural practices that release NO_2_ into the atmosphere [55]. In contrast, we found negative associations with NO_2_ in areas with non-clean energy and forest areas. Recent studies revealed that forest areas can act as natural filters for air pollutants, helping to mitigate the impact of weather changes on air pollution [50]. Concerning the non-clean energy sources, the coefficients increase when the percentile gets higher, suggesting that the negative association mentioned only occurs in regions with little non-clean energy sources.

#### 3.3.3 SO_2_

Sulfur Dioxide is a colorless and non-flammable gas with a distinct odor. This pollutant reacts to the presence of Oxygen to form SO_3_, which dissolves in water to form sulfuric acid (H_2_SO_4_). As seen in NO_2_, their main anthropogenic sources derive from burning and refining fossil fuels, while the main natural sources are volcanoes activities, organic material decay, and solar action on seawater, [39,54,56].

**Fig. 6:**
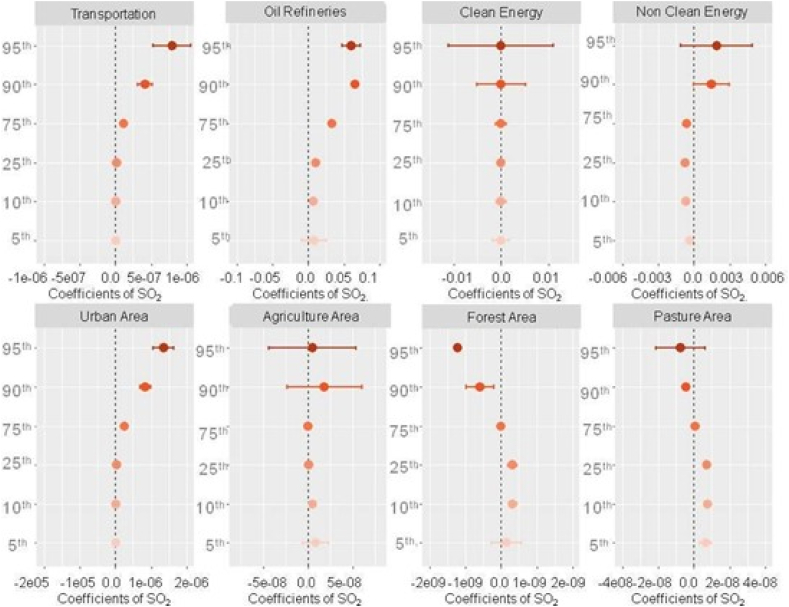
Quantile regression coefficients for weather penalties and land use types/activities factors over SO2 for the period 2003–2018.

Our results show that oil refineries, urban areas, and transportation are the land use variables with the most significant positive influence over the SO_2_ weather penalty. Our findings suggest that the effect of these variables increases as the percentile of the SO_2_ weather penalty increases. At the 95th percentile, the influence of oil refineries, urban areas, and transportation reaches its maximum level, while at the 5th percentile, these variables have their lowest coefficient (Fig. 6). All these variables are directly related to the SO_2_ emission sources [44,54,56], which explains this association.

In contrast, non-renewable energy and forest areas had negative associations (Fig. 6). These results suggest that targeted interventions in oil refineries, urban areas, and transportation may be needed to reduce SO_2_ pollution, particularly during periods of extreme weather conditions. As mentioned earlier, in Brazil, non-renewable energy sources, specifically the thermoelectrical power plants, based on fossil fuel burning, are usually turned on during the dry seasons, increasing the emissions of SO_2_ only in this specific periods. Usually, cities with low quantities of non-clean energy are more dependent on clean sources, which leads to a lower concentration of SO_2_ emissions [57].

#### 3.3.4 O_3_

**Fig. 7:**
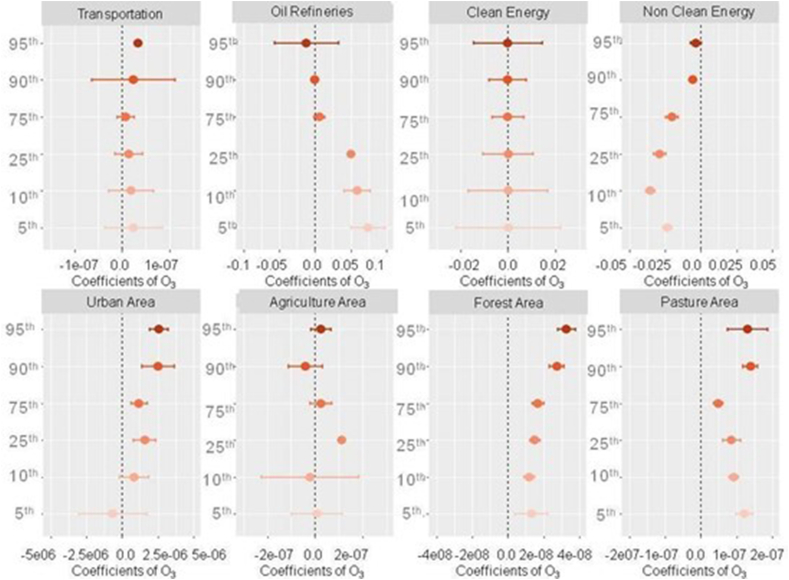
Quantile regression coefficients for weather penalties and land use types/activities factors over O_3_ for the period 2003–2018.

Our results show that the influence of land use types and activities on the association between weather changes and O_3_ varied by percentile of the weather penalties (Fig. 7). Robust positive associations were observed for transportation (only at the 95th percentile), oil refineries (only for the lowest percentiles, <25th), urban areas (excluding the 10th and 5th percentiles; the 95th percentile had the highest effect), forest urban areas (all percentiles), and pasture areas (all percentiles). Notably, the highest effect was observed for urban areas at the 95th percentile. In contrast, non-clean energy had negative associations with weather penalties on O_3_ across all percentiles.

In urban areas, the association between weather and O_3_ is typically positive, meaning that higher temperatures and sunlight intensity can lead to increased O_3_ pollution. This is because urban areas often have high concentrations of NOx emissions from traffic and industrial sources, as well as high concentrations of VOC emissions from various sources [52,53,58]. When these emissions are exposed to sunlight and high temperatures, they can react to form O_3_. Specifically, in areas with traffic (note that transportation had positive associations in our analyses at the 95th percentile), the association between weather and O_3_ is also typically positive. This is because traffic is a major source of NOx emissions, which can react with VOCs in the presence of sunlight and high temperatures to form O_3_ [5,6].

Similarly, in areas with oil refineries, the association between weather and O_3_ is typically positive, as oil refineries can also be a major source of NOx emissions [[59], [60], [61]]. Additionally, oil refineries may release VOCs during certain processes [61], which can also contribute to the formation of O_3_ in the presence of sunlight and high temperatures.

In areas with non-clean energy generation, the association between weather and O_3_ is typically negative, meaning that higher temperatures and sunlight intensity can lead to decreased O_3_ pollution. This is because non-clean energy sources, such as coal-fired power plants, can emit sulfur dioxide (SO_2_) and nitrogen oxides (NOx), which can react with O_3_ to form other pollutants.

In forest areas, there are emissions of VOCs, which can react with NOx emissions from other sources to form O_3_. However, forests can also absorb O_3_ through their leaves and other surfaces, which can lead to decreased O_3_ concentrations. This mix of conditions may explain our results indicating positive associations in forest areas [57,62,63].

In pasture areas, the association between weather and O_3_ is typically positive. This is because pasture areas may have high levels of VOC emissions from vegetation, as well as high concentrations of NOx emissions from agricultural sources. When these emissions are exposed to sunlight and high temperatures, they can react to form O_3_ [57,62,63].

### 3.4 Study limitations

Our study has a few limitations that must be considered to correctly interpret our findings. First, both air pollution and weather data were derived from global datasets and remote sensing models, without ground measuring, which can result in errors since there is a spatial and temporal limitation of the air quality monitoring network in Brazil. On the other hand, the lack of data for some Brazilian regions such as North and Northeast reinforces the relevance of this study.

The validation studies cited earlier, reassure that ECMWF and the CAMS Global model can be a reliable source of providing such data [25]. To minimize this issue and reduce the outlier effect, we removed the values above the 95th percentile and aggregated the datasets into a daily scale for every variable used on the model. Second, we applied a limited set of four weather parameters to assess the impact of climate on air pollution. Other additional weather variables (e.g., radiation, cloud cover and wind direction) should be considered in further studies since they are also associated with air pollutants concentrations. Third, the land use and energy information were considered only for the year 2018, despite the time series of 16 years used for the weather penalty calculation. Finally, the stochastic nature of our methods cannot provide causal explanations for our results.

## 4 Conclusions

Our study aimed to estimate the influence of land use types and activities on the association between weather changes and ambient air pollution in Brazil. Using a two-stage design, we found significant associations between weather changes and air pollution for all four pollutants studied (PM_2.5_, NO_2_, SO_2_, O_3_). Our results suggest that different land use types and activities have varying effects on the association between weather changes and air pollution, highlighting the need for targeted policies and interventions that address local characteristics.

Regarding PM_2.5_, we found that agricultural areas and non-clean energy were positively associated with the weather penalty, while forest areas had a negative association. For NO_2_ and SO_2_, we observed positive associations with transportation, oil refineries, and urban areas, while non-clean energy and forest areas had negative associations. Finally, for O_3_, we found positive associations with transportation, oil refineries, urban areas, forest urban areas, and pasture areas, while non-clean energy had a negative association.

Our study provides important insights into the complex relationship between land use, weather changes, and ambient air pollution.

Municipalities with more forested zones, fewer oil refineries, more clean energy sources, and smaller vehicular fleets are suggested to be less impacted by weather changes concerning their pollution levels.

By associating urban areas, transportation, oil refineries, and non-clean energy sources with an increase in the weather impact on air pollution concentrations, at a municipality level, it revealed a link between local needs such as the development of cleaner transportation systems, enhancement of more green forested zones in urban areas and location of oil refineries with a global scale phenomenon.

In a Climate crisis, these results have significant implications for policymakers and urban planners who seek to mitigate the effects of air pollution in Brazil. This is a critical issue in a country where air pollution has been linked to significant health problems, including premature deaths. By identifying the key drivers of air pollution in different regions of Brazil, our study can inform the development of effective mitigation strategies that protect public health.

## Availability of data and materials

Data associated with the study has not been deposited into a publicly available repository. Data are available from the corresponding author on reasonable request.

## CRediT authorship contribution statement

**Francisco Jablinski Castelhano:** Writing – review & editing, Writing – original draft, Visualization, Validation, Supervision, Software, Methodology, Investigation, Formal analysis, Data curation. **Weeberb J. Réquia:** Writing – review & editing, Software, Resources, Project administration, Methodology, Conceptualization.

## Declaration of competing interest

The authors declare that they have no known competing financial interests or personal relationships that could have appeared to influence the work reported in this paper.
