# Supplementary material for: Weather impact on ambient air pollution and its association with land use types/activities over 5,572 municipalities in Brazil
Source: Heliyon. 2024 May 24;10(11):e31857. doi: 10.1016/j.heliyon.2024.e31857 (PMC11177152; doi:10.1016/j.heliyon.2024.e31857)
Supplement: Multimedia component 1 [file mmc1.docx]

# **Weather impact on ambient air pollution and its association with land use types/activities over 5,572 municipalities in Brazil**

**Supplementary Materials**

**Francisco Jablinski Castelhano**

Geography Department, Federal University of Rio Grande do Norte Natal, Rio Grande do Norte, Brazil

Av. Sen. Salgado Filho, S/n - Lagoa Nova, Natal - RN francisco.castelhano@ufrn.br

+55 41 98816 8885

*Corresponding author

# **Weeberb J. Réquia**

[weeberb.requia@fgv.br](mailto:weeberb.requia@fgv.br)

School of Public Policy and Government, Fundação Getúlio Vargas

Brasília, Distrito Federal, Brazil


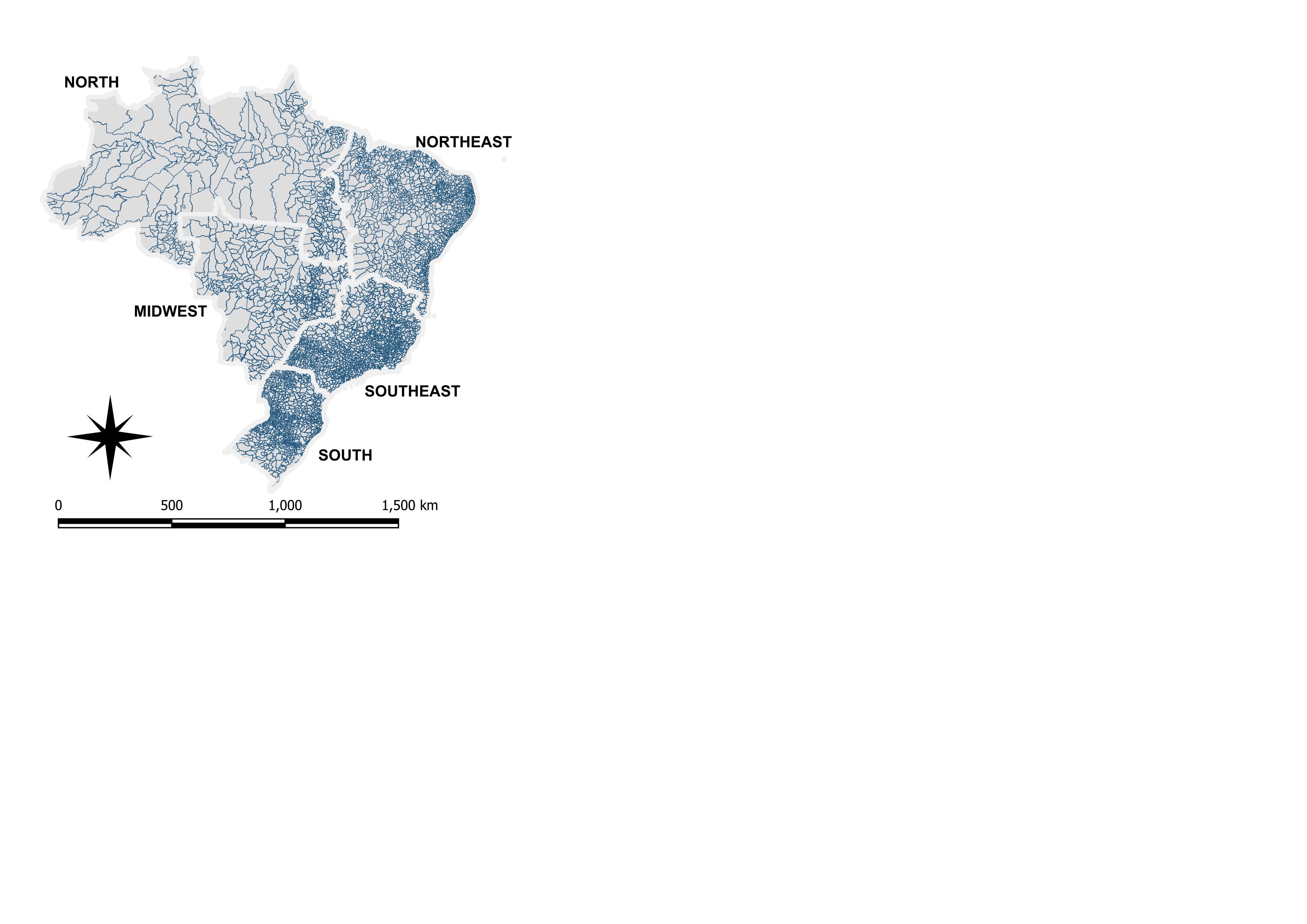


**Supplementary Figure 1 -** Brazil's regions and municipalities.
